# The incidence of kidney cancer in Iran: a systematic review and meta-analysis

**DOI:** 10.1051/bmdcn/2018080209

**Published:** 2018-05-28

**Authors:** Soheil Hassanipour, Gholamreza Namvar, Mohammad Fathalipour, Hamid Salehiniya

**Affiliations:** 1 Gastrointestinal & Liver Diseases Research Center (GLDRC), Guilan University of Medical Sciences Rasht Iran; 2 Student Research Committee, Shiraz University of Medical Sciences Shiraz Iran; 3 Tabriz University of Medical Sciences Tabriz Iran; 4 Zabol University of Medical Sciences Zabol Iran; 5 Department of Epidemiology and Biostatistics, School of Public Health, Tehran University of Medical Sciences Tehran Iran

**Keywords:** Incidence, Kidney cancer, Iran

## Abstract

Background: The incidence of kidney cancer from different areas of Iran was reported. Nevertheless, there is no available systematic reviews in this regard. Therefore, the present systematic review carried out to estimate the incidence rate of kidney cancer among Iranian people.

Method: This systematic review was performed according to the Preferred Reporting Items for Systematic Reviews and Meta-Analysis (PRISMA) in September 2017. A search was concluded using Medline/ PubMed, Scopus, ScienceDirect, and Google scholar for international papers and four national databases (Scientific Information Database, MagIran, IranMedex, and IranDoc) for Persian papers. The incidence rate of kidney cancer was calculated using random effect model.

Result: An aggregate of 159 papers were retrieved in the primary search of the databases. Further screening and advanced refinement of the retrieved studies produced 15 studies totally. The age-standardized rate (ASR) of kidney cancer was 1.94, 95% CI (1.62-2.55) and 1.36, 95 % CI (1.09-1.62) in males and females, respectively.

Conclusion: In comparison to other parts of the world, the incidence of kidney cancer was lower in Iran. Afterwards, further studies are necessary to outline the exact incidence rate and the trend of kidney cancer in Iran.

## 1. Introduction

Cancer is an important cause of death worldwide. Among cancers, Kidney cancer (KC) is known as the most murderous urinary tract cancer and the 9^th^ and the 14^th^ most common cancer for men and women, respectively [1]. Over 300000 (200000 males) new cases of KC have been diagnosed annually with about 140000 (90000 males) deaths [2]. The renal cell carcinoma is more than 90 percent of all kidney that occurs in both genders and the incidence and prevalence of the renal cell carcinoma has been increasing over the times [3].

KC has a different geographical distribution based on population, lifestyle, nutrition, physical activity, and environmental factors. The incidence rates of KC varies more than 15-fold worldwide; Eastern European countries have the highest and South America have the lowest incidence [4]. Although few epidemiological studies have been done on the KC in Iran, it is the most common urological cancer among Iranian men and women [5] and one of top ten cancers in the population of Southern Iran [6].

Formal cancer-related data of Iranian population were published in 1956 for the first time [7]. The National Cancer Registry in Iran was established in 1984. Thereafter, various types of reports were published about the incidence and prevalence of cancers [8-10]. To the best of our knowledge, there is no study on the exact incidence rate of KC among Iranian.

Therefore, we carried out a systematic review and meta-analysis of the Iranian studies to determine the incidence rate estimation of KC among the Iranian population.

## 2. Methods

The study was planned and conducted in 2017. The systematic review was conducted using the Preferred Reporting Items for Systematic Reviews and Meta-Analysis (PRISMA) checklist [11].

### 2.1. Search strategy for systematic reviews

A literature search of published studies was conducted using Medline/PubMed, Scopus, ScienceDirect, Embase and Google Scholar as international databases, and Scientific Information Database (SID) (www.sid.ir), MagIran (www.magiran.com), IranMedex (www.iranmedex.com), and Irandoc (www.irandoc. ac.ir) as national databases in September 2017. No time duration limitation was considered. The keywords included: “kidney cancer”, “kidney neoplasms”, “kidney tumor”,” kidney carcinoma”, “cancer of kidney”, “neoplasms of kidney”, “renal cancer”, “renal cell carcinoma”, “renal neoplasms”, “renal tumor”, “cancer of renal”, “neoplasms of renal”, “incidence”, and “Iran”. The citation results were then imported into EndNote X5 software (Thomson Reuters, Carlsbad, CA, USA). Thereafter, the studies were checked out by two reviewers independently.

### 2.2. Inclusion and exclusion criteria

The papers contained clearly reports of the age-standardized rate (ASR) of KC and obvious description of Iranian populations of any language and time were included. In addition, the papers with the following criteria were excluded: studies which reported prevalence rate based on pathological data, studies with insufficient sample size, the poster and conference papers, and duplicated studies.

### 2.3. Statistical analysis

All the analysis were conducted using STATA software, version 12 (Stata Corp LP, College Station, TX, USA). Statistical heterogeneity was assessed by Cochran’s Q statistic (with a significance level of p ≤ 0.1) and I^2^ statistic (with a significance level of ≥ 50%). In the presence of significant heterogeneity among the studies, the Meta-analysis was done by random effect model (with inverse variance method) was used. On the other hand, in the case which is not heterogeneous (p > 0.1 and I^2^ < 50%), fixed effect model was performed.

## 3. Results

### 3.1. Description of literature search

**Fig. 1:**
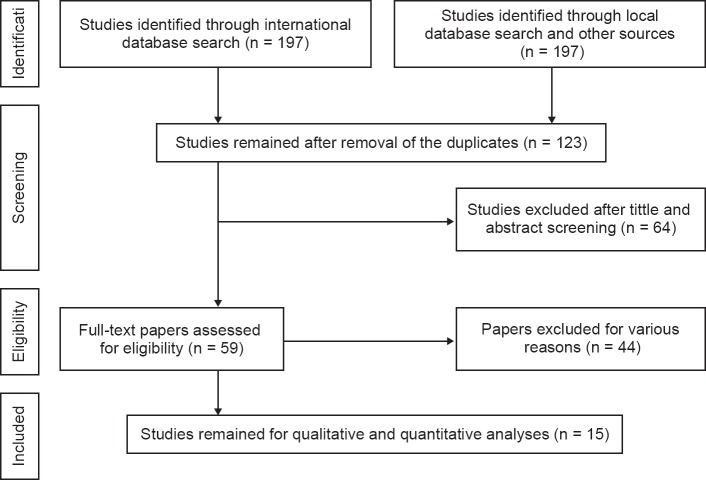
Flowchart of the included eligible studies in the systematic review.

The search process and Study selection base on PRISMA flow chart in this systematic review has been outlined in Fig. 1. The literature searches yielded 159 potentially relevant studies from the primary searches. In total, 98 studies met inclusion criteria and entered into the second stage of evaluation. Some studies were excluded for the following reasons: being irrelevant to the topic (n = 52), incorrect study population (n = 19), duplicate studies (n = 4), and insufficient data (n = 8). Overall, the review included 15 unique studies.

### 3.2. Description of the included studies

The basic characteristics of the included studies have been summarized in Table 1. According to the geographical area, three studies were conducted in Fars province [6, 12, 13], two in Ardabil province [14, 15], one in Kerman province [16], one in Semnan province [17], one in East Azerbaijan province [18], one in Tehran province [19] and one in Shahroud city [20], and five in all states of Iran [21-25].

**Table 1:** Basic characteristics of the studies.

Order	Author, Year	Time period	Location	ASR (Males)	ASR (Females)
1	Sadjadi, 2003	1996-1999	Ardabil	1.10	1.30
2	Babaei, 2004	1996-2000	Semnan	2.27	0.71
3	Sadjadi, 2007	1996-2000	Kerman	0.90	0.50
4	Somi, 2008	2006-2007	East Azarbaijan	3.42	1.76
5	Mehrabian, 2008	1990-2005	Fars	0.91	0.59
6	Babaei, 2009	2000-2004	Ardabil	3.10	2.90
7	Mohagheghi, 2009	1998-2001	Tehran	3.20	1.70
8	Mousavi, 2009	2003-2004 2004-2005 2005-2006	Iran	1.39 1.76 2.12	0.96 1.10 1.41
9	Masoompour, 2011	1998-2002	Fars	1.30	0.70
10	Fateh, 2013	2000-2010	Shahroud	0.79	0.30
11	Basiri, 2014	2003 2009	Iran	1.39 1.97	0.96 1.93
12	Roshandel, 2014	2012	Iran	2.00	1.40
13	Mirzaei, 2015	2003 2004 2005 2006 2007 2008 2009	Iran	1.39 1.76 2.08 2.35 2.43 2.93 2.99	0.96 1.10 1.39 1.58 1.64 1.81 2.05
14	Masoompour, 2016	1985-1989 1998-2002 2007-2010	Fars	0.97 1.30 3.81	- - -
15	Arabsalmani, 2017	2012	Iran	2.10	3.00

### 3.3. The results of individual studies

This study showed the male to female sex ASIR ratio is 1.42. The highest ASR (3.81 per 100,000) of males was reported from Fras province between 2007 and 2010 [6] and for females (2.9 per 100,000) was reported from Ardabil province between 2000 and 2004 [15], while the lowest ASR in males and females was reported from Kerman province between 1996 and 2000 (0.9 and 0.5 per 100,000 for males and females, respectively) [16].

### 3.4. The results of meta-analysis

**Fig. 2:**
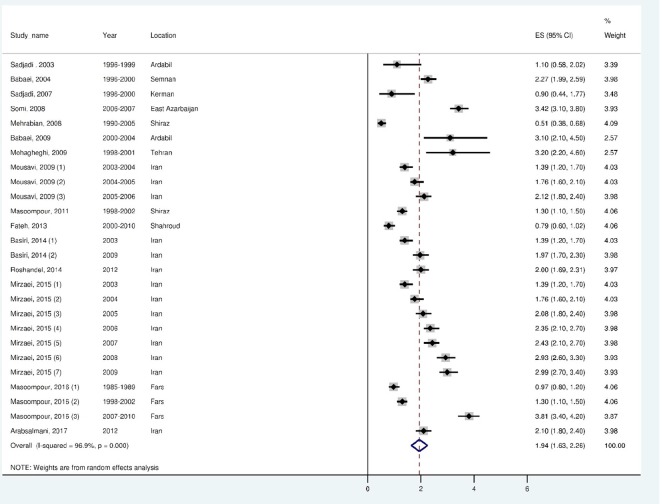
Forest plot of the random-effect meta-analysis for ASRs of kidney cancer in males in Iran.

**Fig. 3:**
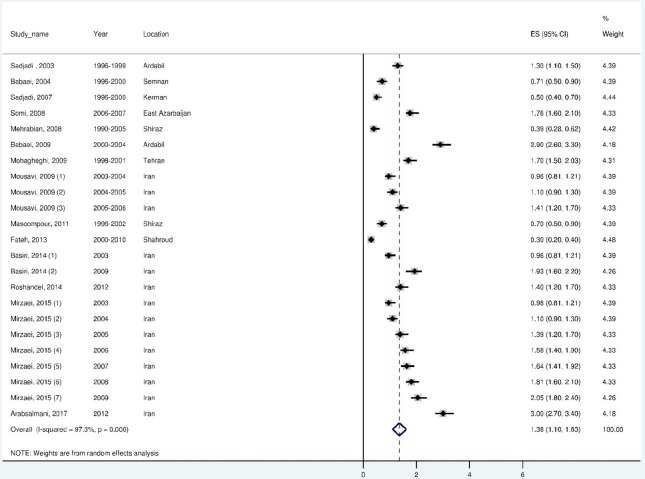
Forest plot of the random-effect meta-analysis for ASRs of kidney cancer in females in Iran.

The ASR of KC was 1.94, 95% CI (1.62 to 2.55) for males and 1.36 95 % CI (1.09 to 1.62) in females. The heterogeneity of the studies was demonstrated by Cochran’s test (Q = 799.6, df = 25, I^2^ = 96.9%, *p* < 0.001) for males and (Q = 807.2, df = 22, I^2^ = 97.3%, *p* < 0.001) for females. The results of the random-effect metaanalysis for ASRs of KC for males in Iran has been represented in Fig. 2 and for females in Fig. 3. All the measurements in the forest plot were magnified by 10^5^.

## 4. Discussion

The aim of the present study was to evaluate the incidence rate of KC in Iran. The results demonstrated that the incidence rate of KC among Iranian men is low (ASR = 1.94 per 100,000). Some Asian countries such as South Korea (4.7 per 100,000), Turkey (4.4 per 100,000), and Mongolia (3.3 per 100,000) have high Standardized Incidence Ratios (SIR). Additionally, other countries such as Indonesia (1 per 100,000), Turkmenistan (1.2 per 100,000) and Kyrgyzstan (1.4 per 100 thousand) have the lowest SIR [25].

The results of the study have demonstrated that the incidence of KC among Iranian women (ASR = 1.36 per 100,000) was lower compared to Iranian men (ASR = 1.94 per 100,000). This difference between ASR of female and male is probably related to the different incidence of KC risk factors in Iranian both men and women.

The known risk factors for the most predominant form of KC, renal cell cancer, include hypertension, smoking, obesity, as well as some other less important factors like familial history of KC, environmental and occupational exposure to genotoxic agents or nephrotoxic agents agents acrylamide, cadmium and trichloroethylene, low physical activity, chronic pharmacotherapy with diuretics and phenacetin, alcohol consumption [1]. Although these factors affect the incidence trend of KC, the relative impact of each factor may vary in different populations.

Previous studies have shown that hypertension is more prevalent among Iranian men then woman and hypertension is one on leading causes on KC [26]. Cigarette smoking, as another risk factor for KC, is more prevalent in Iranian males [27, 28]. Additionally, cadmium levels are higher among Iranian men who are living in industrial areas and have environmental and occupational exposure [29]. Among risk factors of KC, these items may cause of high ASR of KC among Iranian males.

According to the results of the study, the highest ASR of KC among Iranian men is observed in Fars province (3.81 per 100,000) and the highest ASR among Iranian women occurs in Ardabil province (2.9 per 100,000). The high incidence rate of KC in Fars province can be attributed to high prevalence of associated risk factors including low socioeconomic status and environmental exposures in this area [30]. Moreover, hypertension is a common health problem in Fars province which has the highest ASR of KC among Iranian men [30] and hypertension can be a major contributor to the high prevalence of KC in this province [31, 32]. For women, Ardabil province is an area with different disparities in terms of the prevalence of possible risk factors in compare to other provinces in Iran. The high prevalence of KC in this region can be due to different genetic and environmental factors of the area [14, 33].

The results of the current study have shown that the lowest ASR of KC in Iran, in both genders, is observed in Kerman province (0.9 for men and 0.5 for women per 100,000). The low incidence rate of KC in this province can be attributed to the demographic characteristics of the people, differences in lifestyle, and the presence of other types of diseases and cancers [34, 35]. In Kerman province, other types of cancer such as breast, skin, and colorectal cancers among women and skin, bladder, and stomach cancer among men have higher incidence rate [34, 36, 37].

## 5. Conclusion

The incidence rate of KC in Iran was lower in comparison to other parts of the world. Thus, further studies are necessary to outline the exact incidence rate and the trend of KC in Iran.

## Conflict of interest

None declared.
